# Gene expression of carbonic anhydrase 9 (CA9) in *de novo* acute leukemia as a predictive marker for prognosis

**DOI:** 10.25122/jml-2021-0212

**Published:** 2022-09

**Authors:** Kifah Jabbar Alyaqubi, Rasha Hatem Dosh, Rawaa Behlul Al-Fatlawi, Siham Mahmood Al-Rehemi, Najah Rayish Hadi

**Affiliations:** 1 Middle Euphrates of Cancer Research Unit, Faculty of Medicine, University of Kufa, Kufa, Iraq; 2 Department of Human Anatomy, Faculty of Medicine, University of Kufa, Kufa, Iraq; 3 Department of Clinical Laboratory Sciences, Faculty of Pharmacy, University of Kufa, Kufa, Iraq; 4 Department of Pharmacology and Therapeutics, Faculty of Medicine, University of Kufa, Kufa, Iraq

**Keywords:** AML, ALL, CA9, HIF-1α, hypoxia

## Abstract

Carbonic anhydrase 9 (CA9) is a marker for decreased O_2_ concentration and acidosis, associated with poor prognosis in cancerous patients. The current study suggested that the changes in CA9 gene expression level might be used as a predictive marker to assess early prognosis at the time of detection of *de novo* leukemia, and then monitor tumor progress during treatment. This study highlights the level of CA9 gene expression in leukemic patients. A total of 44 cases (acute myeloid leukaemia (AML) group: 23 cases; acute lymphoid leukaemia (ALL) group: 13 cases; control group: 8 healthy volunteers) were selected for this study. The CA9 gene expression was assessed by a real-time PCR with the SYBR green assay. A high level of CA9 gene expression was noticed in AML patients compared to the control group, while the results were not significant in ALL patients. After treatment follow-up, significant differences were observed in CA9 gene expression between a complete response and no response in AML patients. As a result, the CA9 tumor gene could act as a potential early marker for acute leukemia prognosis. A low level of CA9 expression was associated with better clinical outcomes, while a high level was related to a negative prognosis in patients with AML.

## INTRODUCTION

Acute leukaemia is a malignant disease identified by the accumulation of immature hematopoietic cells, called blasts, in the peripheral blood and bone marrow [1, 2]. The most prevalent and heterogeneous type of acute leukaemia in adults is acute myeloid leukaemia (AML) which is a cause of high mortality [3], whereas acute lymphoid leukaemia (ALL) is the most common in children [4]. From 2013 to 2017, the United States registered 4.3 and 1.7 new cases per 100,000 people per year of AML and ALL, respectively, while the cancer death rates (2014–2018) were 2.8 and 0.4 per 100.000 people per year of AML and ALL, respectively [5]. In Iraq, leukaemia was the third most common cancer in both sexes, after breast and lung cancer. In 2018, approximately 1674 new cases of leukaemia and 1,327 deaths from the disease were registered [5].

The tumour environment was correlated to chemotherapy and radiotherapy resistance and contributed to aggressive tumour phenotype [6]. Acute leukaemia screening at early stages could efficiently decrease acute leukaemia-related mortality and prevalence. To prevent acute leukaemia, it is necessary to better comprehend the biology, pathogenesis, genetics and therapy response of leukemic cells. In advanced stages of leukaemia, the bone marrow becomes very hypoxic. Identifying a specific gene expression plays an important role as a predictor of prognosis and therapy response in haematological malignancies [7]. Hypoxia-related genes have recently been identified as possible candidates for prognostic prediction in various human cancers. For instance, hypoxia-inducible factor 1 α (HIF-1α) is considered a marker for hypoxia and plays a vital role in the progression of acute leukemia [8].

In acute myeloid leukemia, the hypoxia-inducible factor-1 (HIF-1) targeting gene CA9 was associated with the clinical outcome [1]. Carbonic anhydrase 9 (CA9) is a transmembrane-related metalloenzyme that catalyzes the reversible hydration of carbon dioxide to bicarbonate and protons, situated on chromosome 9p12-13, which includes 11 exons and encodes for the 459 amino acid proteins. The CA9 is an endogenous marker for malignancy hypoxia [9], which regulates cellular PH in a hypoxic microenvironment and stimulates tumour cell proliferation by enhancing tumour acidosis [9–11]. Furthermore, CA9 has a drug targeting role due to its low expression in normal tissues and increased expression in several cancer tissues, including lung [12], colorectal [13], oral [14], uterine [15], renal [16] and bladder cancer [17]. In addition, CA9 expression was a predictive marker in oesophageal and gastric adenocarcinomas [18].

However, there have been few debatable studies in acute leukaemia, which prompted us to examine the hypoxia-related gene CA9 expression in acute leukaemia patients.The association between CA9 expression and prognosis remains a concern. Thus, in the current study, the gene expression of carbonic anhydrase 9 (CA9) in acute leukaemia was analyzed to investigate whether it represents a promising new target for future therapeutic approaches.

## MATERIAL AND METHODS

Peripheral blood samples were collected from 36 newly diagnosed acute leukemia patients and 8 healthy controls for CA9 investigation. Twenty-three of those patients had AML, and thirteen had ALL. The average percentage of blast cells in the bone marrow and peripheral blood was 67% and 77.7%, respectively. The average age of the patients was 36.8±15.99 years (ranging from 16 to 72 years).

Patient clinical data like blast (%) in the bone marrow and peripheral blood, WBC count, platelet count, hemoglobin, non-response (NR), and complete response (CR) were documented from the tumor registry files with the assistance of medical hematologists during follow-up. All patients treated according to the chemotherapy protocols of Baghdad Teaching Hospital-Hematology Unit showed variable responses. Each patient undertook two induction cycles followed by consolidation. Response to treatment classified as complete response (CR) was maintained for more than 6 months. The percentage of blast cells was less than 5%, the cellularity was more than 20% in the bone marrow aspirate after induction of chemotherapy, and there was an absence of leukemia in other locations. In non-response (NR) patients, there were more than 5% of blast cells in the bone marrow, and leukemia in other locations was indicated after at least two courses of chemotherapy. CR and NR were recorded after each cycle of induction.

### Samples preservation

The blood samples were well-kept in TRIzol at the genetic laboratory of the National Center for Initial Diagnosis of Tumors in the medical city of Baghdad/Iraq. 0.5 ml out of 2 ml of peripheral blood was conserved as whole blood. Samples were centrifuged at 1.000 xg for 5 minutes at 4℃, then the supernatant was removed, and 5% Triton X-100 was added to phosphate buffer saline (PBS) and mixed with the samples to be homogenized. TRIzol (0.75 ml) was added to each sample in a part of 3 TRIzol: 1 sample. Lastly, the samples were kept at a temperature of -80℃. RNA was extracted from the samples. The molecular study was conducted using reverse transcription and real-time PCR at the molecular oncology unit from Guy's Hospital King's College London, UK.

### RNA extraction, reverse transcription and real-time -PCR assay

The TRIzol® LS Reagent (Life Technologies-Ambion USA) was used to achieve total RNA extraction from all groups of blood samples following the producer's protocol. By using High-Capacity cDNA Reverse Transcription Kit (Life Technologies/Ambion/USA), reverse transcription of total RNA was shown in a reaction volume of 20 µl (15 µl total RNA, 2 µl RT buffer, 0.2 µl RT random primers, 0.8 µldNTPs mix, 1 µl RNase inhibitor, and 1 µl reverse transcriptase). As a final point, cDNA was held in reserve at -80C° until used. The gene expression was evaluated using certain primers designed with Primer 3 (http://www.ncbi.nlm.nih.gov/tools/primer-blast) (Table 1). To make the standard curve, serial dilutions of cDNA were used. The standard curves were produced for the target gene and endogenous control gene (ABL). Applied-Biosystems 7900 real-time PCR machine was used for quantitative real-time PCR assays in triplicate. The 20 microliters of reaction volume contained 1 µl of primer mixes, 10 µl of SYBR Green master mix, 4 µl of cDNA template, and 5 µl of RNase-free water. Real-Time PCR protocol was as follows: step 1: 50℃ for 2 minutes, step 2: 95℃ for 10 minutes, step 3, in a two-step cycle: 95℃ for 15 seconds and 65℃ for 1 minute repeated for 6 cycles, and step 4 in a two-step cycle process: 95℃ for 15 seconds and annealing at 61℃ for 1 minute repeated for 40 cycles.

**Table 1:** Primers sequences.

Primer	Sequence
CA9-F	5'-GTGGAAGGCCACCGTTTC-3'
CA9-R	5'-CTCGTCAACTCTGGCAAAGG-3'
ABL-F	5'-TGGAGATAACACTCTAAGCATAACTAAAGGT-3'
ABL-R	5'-GATGTAGTTGCTTGGGACCCA-3'

### Quantitative real-time polymerase chain reaction data analysis

The volume of the target CA9 gene was normalized to an endogenous reference ABL gene. Relative to a calibrator, untreated normal control was given by: (2-∆∆Ct) ABI PRISM 7700 Sequence Detection System 1997 (User- Bulletin No.2,1997). To evaluate the level of expression for different blood samples, the relative expression level was calculated using the comparative CT method (threshold cycle) and compared with a calibrator. The comparative CT method removes the use of standard curves for relative quantitation when the PCR efficiency of the target and reference gene is similar. The gene expression fold change was calculated by (2-∆∆Ct) where ∆∆Ct=∆Ct target- ∆Ct untreated for calibration and normalized by ∆Ct=Ct target gene- Ct endogenous reference.

### Relative efficiency of CA9 and ABL genes

In order for the ΔΔCT calculation to be effective, the efficiency of the reference amplification and the efficiency of the target amplification must be roughly equal to ABI PRISM 7700 Sequence Detection System 1997 (User Bulletin No.2,1997). The efficiency of the reference amplification and the target amplification was calculated using a standard curve for CA9 and ABL necessary data according to the equation E=10^(-1/slop)^.

### Statistical analysis

SPSS program version 20 was used to analyze the data. Categorical variables were represented as frequencies and percentages. Shapiro-Wilk test was significant for CA9 gene expression, which indicated that it is not normally distributed. So, the Mann-Whitney U test was used for comparisons between two groups and the Kruskal-Wallis test was used for comparison among all study groups. Statistical significance was accepted at P≤0.05.

## RESULTS

A total number of thirty-six *de novo* acute leukemia patients and eight healthy volunteers as a control group were characterized in Table 2.

**Table 2:** Descriptive characteristics of control and leukaemia patients.

Variable		No.	%	Blast Average %
**Groups**	AML	23	52.3	62% (ranged 15–97)
	ALL	13	29.5	59% (ranged 3–98)
	Control	8	18.2	
**Gender**	Male	23	52.3	
	Female	21	47.7	
**Age group (years)**	16–19	9	20.5	
	20–29	11	25.0	
	30–39	7	15.9	
	40–49	10	22.7	
	50+	7	15.9	

**Figure 1:**
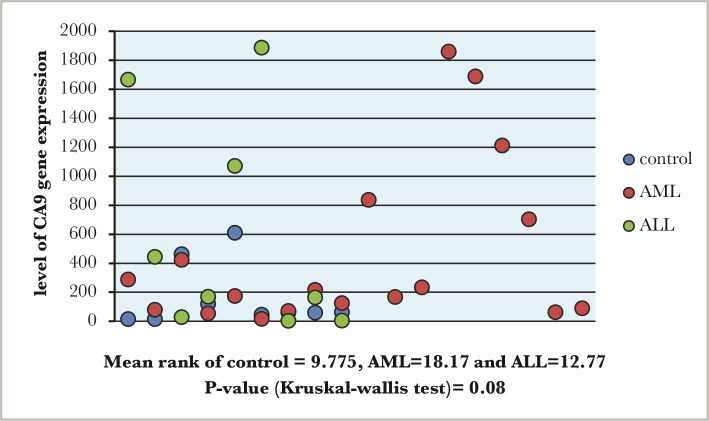
The comparison of CA9 expression among study groups.

The comparison between acute lymphoblastic leukemia (n=13, mean rank 12.77), acute myeloblastic leukemia (n=23, mean rank 18.17) and the control group (n-8, mean rank 9.75) showed no significant differences in the level of CA9 gene expression (p value=0.08) (Figure 1).

**Figure 2:**
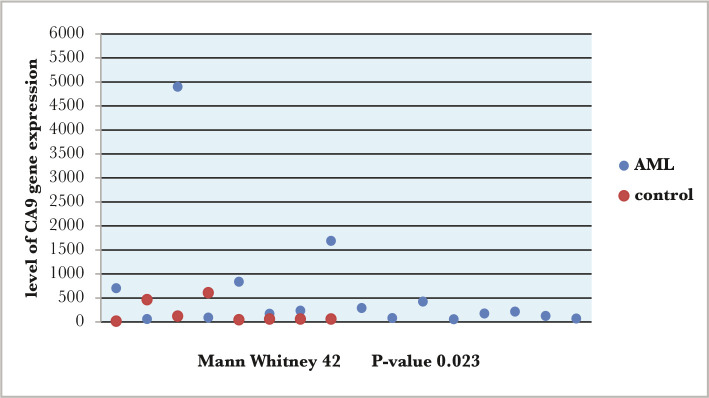
Comparisons between AML patients and control group according to the level of CA9 gene expression.

**Figure 3:**
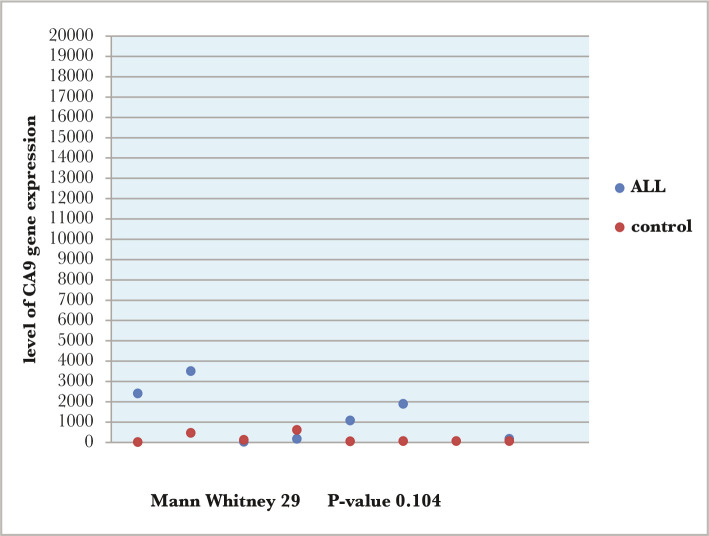
Comparisons between ALL patients and the control group according to the level of CA9 gene expression.

As for the CA9 gene expression in AML patients, the results showed a significant difference from the control group (p-value=0.023) (Figure 2). However, CA9 gene expression in the ALL group showed no significant difference from the control group (p-value=0.104) (Figure 3).

**Figure 4:**
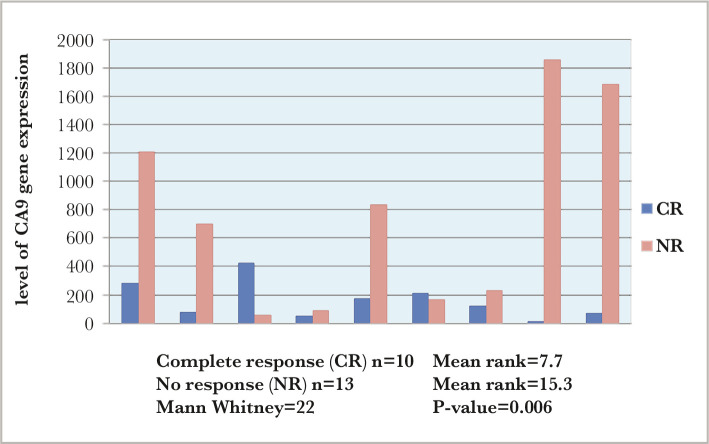
The level of CA9 gene expression in CR and NR AML patients.

During the treatment follow-up, there were highly significant differences between the level of CA9 gene expression in complete response (CR) and non-response (NR) AML patients conferring to a mean rank of 7.7 CR and 15.3 NR (p-value=0.006) (Figure 4). While in ALL patients, the results showed no significant differences in clinical outcomes (p-value=0.093) (Table 3).

**Table 3:** The level of CA9 gene expression in CR and NR ALL patients.

Type of leukemia	Response	Mean rank	Mann Whitney	P-value
**ALL**	Complete response (n=5)	4.6	8	0.093
	No response (n=8)	8.5	-	

## DISCUSSION

Carbonic anhydrases have been a target for research in tumour invasion and carcinogenesis. Carbonic anhydrase 9 (CA9) is a transcription factor that responds to a reduction in the oxygen supply in the tumour microenvironment. As a result, CA9 is up-regulated under hypoxia [19, 20]. CA9 is over-expressed in many epithelial neoplasms and has been linked to tumour hypoxia and carcinogenesis [21]. CA9 is a hypoxia-related gene identified with prognostic value in patients with AML. Continuous CA9-expression is an indicator of poor prognosis not only in solid tumors but also in hematological cancers [22]. In specific types of leukaemia, CA9 is up-regulated, which may control neo-angiogenic pathways [23]. In this study, the expression of CA9 was assessed in the peripheral blood samples of 36 leukemic patients and 8 healthy volunteers. The comparison between AML and the control group showed a significant difference in CA9 expression (p=0.023), while the observed difference between ALL and the control group was not significant. The microenvironment of the bone marrow (BM) controls the survival and normal growth of hematopoietic stem cells. Hypoxia is vital for the survival of the quiescent hematopoietic stem cell in the niche. The BM is extremely hypoxic, and disease development is related to the increased niche hypoxia [20]. The interactions between leukemia cells and their microenvironment in the bone marrow enhance cell viability and increase resistance to anti-leukemic treatments. The hypoxic environment of the leukemic bone marrow provided a reason for targeting it. Our results agree with Benito et al. (2011) who demonstrated that targeting hypoxia is possible and could have a massive effect on leukemia treatment. They showed that HIF-1a positivity in the bone marrow of ALL patients was extremely high at diagnosis, but it was dramatically decreased or removed once the patients reached CR. Their justification was that the aggregation of leukemic blasts in the bone marrow causes increased oxygen intake, therefore reducing steady-state oxygen concentration [24]. In accordance with our results, a previous *in vitro* experimental study demonstrated that CA9 was up-regulated under hypoxic conditions in the cell line of oesophageal squamous cell carcinoma [18]. In addition, another study of a mouse model with squamous and islet cell carcinoma reported that the inhibition of HIFs alpha delays the progression of leukaemia by suppressing cell migration, neoangiogenesis, and colony formation [25].

CA9 is commonly related to tumour radio and chemoresistance due to its role in maintaining cellular homeostasis in hypoxic conditions, allowing tumour cell detection and, as a result, aggressive tumour phenotypic traits [26]. Hypoxia in the bone marrow plays a role in AML blast chemoresistance, and as a result, it can activate NF-kB, which regulates essential pathways such as cell proliferation, metastasis, angiogenesis and survival. The NF-kB pathway was modulated to make leukemic cells more responsive to chemotherapy and to prevent leukaemia cell growth [27].

CA9 is a predictive indicator of survival in patients with leukaemia, colorectal cancer [28] and urothelial cell carcinoma [29]. Resistance and relapse in many forms of cancer are believed to be caused by a limited population of stem-like cancer cells, called haematological cancer stem cells, with the ability of self-renewal and differentiation. These cells are active in the initiation and maintenance of hematological cancers [22]. This study supports evidence from previous observations that AML is the most frequent leukaemia in adults. In younger patients (less than 60 years), the complete remission (CR) rate is between 65% and 75% after intensive induction therapy. However, over half of these patients will relapse. Consequently, the overall survival rate after five years is 30% to 40% [30, 31], whereas, in patients older than 60, the statistics are more unfavourable. Appelbaum et al. observed that 33% of AML in patients (<60 years) and 57% in patients (>75 years) were resistant to treatment [32]. Immunotherapeutic methods targeting tumour-associated antigens (TAAs) to prevent relapses could offer a promising new treatment to improve the recovery of AML patients [32]. Drolle et al. reported that internal tandem duplication (ITD) of the fetal liver tyrosine kinase 3 (FLT3) is clearly associated with a poor prognosis. The existence of ITD has no effect on achieving complete remission, but it does increase the risk of relapse, and tyrosine kinase inhibitors have only a moderate effect [33]. As a result, factors (cellular and non-cellular components of the microenvironment) other than blast mutations influence leukemic cell activity *in vivo* [34].

## CONCLUSION

In conclusion, the present study demonstrates that CA9 gene expression shows a correlation with AML growth and survival. Controlling CA9, which is induced under hypoxic conditions, is critical for improving the outcomes of cancer therapy. CA9 inhibition has been proposed as a way to increase the efficiency of chemotherapy and radiotherapy. Despite these promising results, further immunohistochemistry staining for detecting CA9 in leukaemia samples is recommended.

## Personal thanks

Special thanks to the genetic laboratory of the National Lab in the Medical City Teaching Hospital Baghdad/Iraq ,and the Molecular Oncology Unit, Guy’s Hospital Kings College University London.
